# Inhibition of XPO1 by selinexor enhances terminal erythroid maturation through modulation of HSP70 trafficking in severe β^0^-thalassemia/HbE

**DOI:** 10.1371/journal.pone.0333127

**Published:** 2025-09-25

**Authors:** Pinyaphat Khamphikham, Adisak Tantiworawit, Songyot Anuchapreeda

**Affiliations:** 1 Division of Clinical Microscopy, Department of Medical Technology, Faculty of Associated Medical Sciences, Chiang Mai University, Chiang Mai, Thailand; 2 Hematology and Health Technology Research Center, Department of Medical Technology, Faculty of Associated Medical Sciences, Chiang Mai University, Chiang Mai, Thailand; 3 Division of Hematology, Department of Internal Medicine, Faculty of Medicine, Chiang Mai University, Chiang Mai, Thailand; Imam Abdulrahman Bin Faisal University, SAUDI ARABIA

## Abstract

Ineffective erythropoiesis is a hallmark of β-thalassemia, characterized by impaired erythroid maturation and increased apoptosis of erythroid precursors in the bone marrow, resulting in chronic anemia. Heat shock protein 70 (HSP70) trafficking has emerged as a critical regulator of erythroid maturation. Inhibition of nuclear export protein exportin-1 (XPO1) retains HSP70 in the nucleus, thereby promoting terminal erythroid maturation (TEM) through stabilization of the transcription factor GATA1. In this study, we screened nine XPO1 inhibitors, including the natural compounds curcumin, piperlongumine, plumbagin, and oridonin, as well as the synthetic agents KPT-185, KPT-276, selinexor, verdinexor, and eltanexor, in erythroid progenitors from patients with severe β^0^-thalassemia/HbE to identify the most effective inducer of TEM and to investigate the downstream molecular mechanisms involved. Selinexor, an FDA-approved drug for multiple myeloma, showed the greatest efficacy in enhancing TEM across nine independent patient samples without altering hemoglobin composition. Combination treatments with hydroxyurea (a γ-globin inducer) and SIS3 (a SMAD3 inhibitor) confirmed selinexor’s dominant effect. Mechanistically, selinexor-induced TEM was associated not only with stabilization of nuclear HSP70 and GATA1 but also with a dose-dependent increase in cytoplasmic HSP70. These findings suggest that cytoplasmic HSP70 trafficking may contribute to erythroid maturation in severe β^0^-thalassemia/HbE, implicating regulatory pathways beyond nuclear GATA1 stabilization. Collectively, our findings highlight the therapeutic potential of repurposing selinexor to enhance erythroid maturation in β-thalassemia and suggest that cytoplasmic HSP70 trafficking warrants further investigation as a contributor to terminal erythroid maturation in β-thalassemia.

## Introduction

β-thalassemia is a major global hematologic disorder, particularly prevalent in Southeast Asia, caused by autosomal recessive mutations in the β-globin gene [1–3]. The coinheritance of β^0^-thalassemia mutations and hemoglobin E variant (*HBB*:c.79G > A), hereafter referred to as β^0^-thalassemia/HbE, constitutes the most prevalent severe β-thalassemia genotype, affecting approximately one million individuals worldwide [1,4,5]. These genetic abnormalities result in insufficient production of adult hemoglobin (HbA, α_2_β_2_) in erythroid cells due to the absence of normal β-globin chains. Consequently, excess α-globin chains accumulate within the cytoplasm of erythroid progenitors in the bone marrow, leading to cellular toxicity, apoptosis, and ultimately ineffective erythropoiesis [2,6]. Patients therefore experience chronic anemia due to reduced HbA levels and dysfunctional red blood cells.

Lifelong blood transfusions combined with iron chelation remain the standard therapy to alleviate anemia in patients with β-thalassemia, but repeated transfusions often result in adverse outcomes, including alloimmunization against minor blood group antigens that complicate donor matching [2,6,7]. Hematopoietic stem cell transplantation is the only established curative option; however, its applicability is restricted by donor availability and transplant-related complications. More recently, gene therapy has emerged as a promising alternative, but accessibility and long-term safety remain important concerns.

Beyond these, additional curative strategies have been explored. One approach aims to mitigate excess α-globin toxicity by reactivating γ-globin expression postnatally, thereby restoring globin balance through increased production of fetal hemoglobin (HbF, α_2_γ_2_) [8,9]. Another strategy involves promoting erythroid survival and maturation, for example by inhibiting the SMAD2/3 pathway downstream of TGF-β signaling [10,11]. While both approaches have shown encouraging efficacy, HbF induction yields variable responses among patients and may not fully correct anemia, whereas enhancing erythroid survival alone may be insufficient to overcome the underlying globin chain imbalance. These limitations underscore the need to identify complementary molecular targets that support the development of next-generation therapies.

Ineffective erythropoiesis is a central pathological feature of β-thalassemia, driven by impaired erythroid maturation and excessive apoptosis of erythroid precursors in the bone marrow. One of the key molecular pathways implicated in this process involves heat shock protein 70 (HSP70), the master erythroid transcription factor GATA binding protein 1 (GATA1), and the nuclear export protein exportin-1 (XPO1). Under physiological conditions, HSP70 stabilizes GATA1 in the nucleus of erythroid progenitors, thereby preserving its function as the central regulator of erythropoiesis [12]. In β-thalassemia, however, excess unpaired α-globin chains sequester HSP70 in the cytoplasm, diminishing its nuclear availability and leaving GATA1 vulnerable to cleavage and degradation, which exacerbates ineffective erythropoiesis [13]. XPO1 has been identified as the nuclear exporter of HSP70, and pharmacologic inhibition of XPO1 (e.g., with KPT-251) has been shown to retain HSP70 in the nucleus, stabilize GATA1, and promote erythroid maturation in culture systems [12,14].

Based on this rationale, we systematically evaluated nine XPO1 inhibitors in an in vitro model of severe β^0^-thalassemia/HbE, including four natural compounds (curcumin, oridonin, piperlongumine, and plumbagin) [15,16] and five synthetic agents (KPT-185, KPT-276, selinexor (KPT-330), verdinexor (KPT-335), and eltanexor (KPT-8602)) [17,18]. The most effective compound, selinexor, was advanced for detailed mechanistic studies of the XPO1-HSP70-GATA1 axis. For comparison, we also investigated hydroxyurea, a well-established HbF inducer, and SIS3, a selective SMAD3 inhibitor, to benchmark selinexor’s efficacy against existing experimental strategies. Our findings reveal an alternative mode of HSP70 trafficking in β^0^-thalassemia/HbE, in which stabilization of nuclear HSP70 and GATA1, together with an induced increase in cytoplasmic HSP70, contribute positively to erythroid maturation.

## Materials and Methods

### Subjects

This study was approved by the Research Ethics Committee, Faculty of Associated Medical Sciences, Chiang Mai University (Study code: AMSEC-65EX-032) and the Faculty of Medicine, Chiang Mai University (Study code: MED-2565-09148). The research was conducted in accordance with the Declaration of Helsinki. Eligibility criteria required a confirmed molecular diagnosis of β^0^-thalassemia/HbE, age between 18 and 50 years, absence of common α-thalassemia deletions (-α^3.7^, -α^4.2^, --^SEA^, and --^THAI^), and no pharmacologic treatments other than blood transfusion within 120 days prior to enrollment. All samples were collected with written informed consent during the periods 01/08/2022–30/06/2023 (AMSEC-65EX-032) and 01/12/2022–31/10/2024 (MED-2565-09148). All participants in this study are described in S1 Table. Disease severity of β^0^-thalassemia/HbE patients was classified using a scoring system as described in a previous study [19]. Informed consent was obtained from all participants prior to the collection of whole blood from a peripheral vein of each volunteer, which was then preserved in an anticoagulant citrate phosphate dextrose adenine solution. Hematological parameters were obtained using Sysmex XN-1000 analyzer (Kobe, Japan).

### Erythroid cell culture

Peripheral blood mononuclear cells (PBMCs) were collected from 25 mL whole blood from participants using Lymphoprep gradient centrifugation (Serumwerk, Bernburg, Germany). CD34 ^+^ hematopoietic stem/progenitor cells (HSPCs) were isolated from the PBMCs using CD34 MicroBead Kit and LS Columns (Miltenyi Biotec, Bergisch Gladbach, Germany). Isolated cells were cultured in a 3-phase erythroid differentiation medium (EDM) for 14 consecutive days as described in a previous study [20]. Briefly, Iscove’s Modified Dulbecco’s Medium (IMDM; Cytiva, South Logan, UT) supplemented with 20% v/v fetal bovine serum, 100 U/mL penicillin and 100 μg/mL streptomycin (Gibco, Grand Island, NY), and 300 μg/mL human holo-transferrin (ProSpec; Ness Ziona, Israel) was prepared as a basal medium. During days 0–4 of culture, cells were seeded in EDM phase I, where the basal medium was supplemented with 50 ng/mL human SCF recombinant protein (SCF; PeproTech, Rehovot, Israel), 10 ng/mL human IL-3 recombinant protein (PeproTech), and 2 U/mL erythropoietin (EPO; Cilag AG, Schaffhausen, Switzerland). During days 4–8 of culture, cells were transferred to EDM phase II, where the basal medium was supplemented with 10 ng/mL SCF and 2 U/mL EPO. During days 8–14 of culture, cells were cultivated in EDM phase III, where the basal medium was supplemented with 4 U/mL EPO. Cell density was maintained at no greater than 1x10^6^ cells/mL and incubated at 37°C, 5% CO_2_, in a humidified condition.

### Compound treatment and Cell assays

Curcumin (02643-14) was purchased from Nacalai Tesque (Kyoto, Japan). Piperlongumine (S7551), plumbagin (S4777), oridonin (NSC-250682; S2335), KPT-185 (S7125), KPT-276 (S7251), selinexor (KPT-330; S7252), verdinexor (KPT-335; S7707), eltanexor (KPT-8602; S8397), and the specific SMAD3 inhibitor SIS3 (S7959) were obtained from SelleckChem (Houston, TX). Hydroxyurea (H8627), a well-established HbF-inducing agent, was purchased from Sigma-Aldrich (St. Louis, MO). All compounds were freshly prepared in dimethyl sulfoxide (DMSO; D8418; Sigma-Aldrich) according to the manufacturer’s instructions. On day 4 of culture (corresponding to EDM phase II), compounds were added either individually or in combination to cells (2x10^5^ cells per condition) at the indicated concentrations. Concentration-matched vehicle controls were prepared using 0.1% v/v DMSO. For cell viability and proliferation assays, cells were harvested and stained with 0.4% trypan blue solution (Gibco). Cell counts were performed using an improved Neubauer chamber under a light microscope. Viability was determined by dye exclusion and expressed as the percentage of live cells within the total population. Proliferation was assessed as the relative increase in viable cell numbers over time.

### Flow cytometry

Cell pellet (2x10^5^ cells) was collected and stained with a PE-conjugated anti-human transferrin receptor (CD71) (CY1G4; BioLegend, San Diego, CA) and an APC-conjugated anti-human glycophorin A (GPA or CD235a) (HIR2; BioLegend) according to the manufacturer’s instructions. Stained cells were analyzed by DxFLEX flow cytometer (Beckman Coulter, Brea, CA) and gated into three regional populations according to the accumulation of CD71 and GPA including R1 (CD71^High^/GPA^High^), R2 (CD71^Medium^/GPA^High^), and R3 (CD71^Low^/GPA^High^). Data from the flow cytometry were analyzed using FlowJo Software version 10.10.0 (BD Life Sciences, Ashland, OR).

### Terminal erythroid maturation (TEM) assessment

Cell pellet (2x10^5^ cells) was collected and loaded into a cytospin apparatus, then transferred onto glass slides at 600 rpm for 4 minutes using a cytocentrifuge (Cytospin 3; Thermo Shandon, Cheshire, UK). Cells were fixed with absolute methanol and stained with Wright-Giemsa solution. Stained cytospins were examined and imaged under a light microscope (Leica DM750; Leica Microsystems, Heerbrugg, Switzerland). To evaluate terminal erythroid maturation (TEM), cytospins were prepared as blinded samples by an independent investigator. Erythroid maturation stages were assessed by a medical technologist based on counts of 100 cells per condition performed in triplicate (S2 Table), and the results are presented as the mean percentage of differential counts (S3 Table). The TEM ratio was calculated as the sum of orthochromatic erythroblasts (Orth) and enucleated erythroid cells (Enuc), divided by the combined counts of basophilic (Baso) and polychromatic erythroblasts (Poly), and expressed as fold change relative to the DMSO control.

### Western blot analysis

Cell pellet (at least 5x10^6^ cells) was collected and subjected to cytoplasmic and nuclear protein extraction using NE-PER Nuclear and Cytoplasmic Extraction Reagents (Thermo Scientific, Rockford, IL) according to the manufacturer’s instructions. Protein concentration was evaluated using Pierce BCA Protein Assay Kit (Thermo Scientific) as per the manufacturer’s protocols. A total of 5−10 μg of protein fractions was separated using tris-glycine SDS-PAGE and transferred onto a PVDF membrane. The membrane was blocked overnight using 5%w/v skimmed milk in 1XPBST (Himedia, Maharashtra, India) prior to immunoblotting. Target proteins were detected using human-specific primary antibodies against exportin-1/CRM1 (#46249; Cell Signaling, Danvers, MA), HSP70 (#4876, Cell Signaling), GATA-1 (#4591; Cell Signaling), lamin A/C (MANLAC1(4A7); DSHB, Iowa City, IA), and GAPDH (ABS16; Merck KGaA, Darmstadt, Germany), and HRP-conjugated species-specific secondary antibodies including goat anti-rabbit IgG (W401B; Promega, Madison, WI) and goat anti-mouse IgG (W402B; Promega). Chemiluminescent signals were developed using Immobilon Forte Western HRP substrate (Millipore, Burlington, MA) and detected by exposing to X-ray films. Cytoplasmic and nuclear protein bands were quantified against the band intensity of GAPDH and lamin A/C, respectively, using ImageJ Software.

### Quantitative real-time PCR

Total RNA was extracted from at least 5x10^5^ cells using the RNeasy Mini Kit (Qiagen, Hilden, Germany) following the manufacturer’s instructions. cDNA was synthesized through reverse transcription using the FastKing RT Kit (with gDNase) (Tiangen, Beijing, China) according to the manufacturer’s protocols. Quantitative real-time PCR was performed using mRNA-specific primers (S4 Table) and FastReal qPCR PreMix (SYBR Green) (Tiangen) on the CFX Opus 96 Real-Time PCR System (Biorad, Hercules, CA). Relative gene expression was normalized to ribosomal protein S18 (*RPS18*) expression and calculated using the 2^-ΔΔCt^ method [21].

### Hemoglobin analysis

Cell pellet (at least 1x10^6^ cells) was collected and completely lysed with 500 μL Wash/Diluent Solution (Biorad). The hemolysate was transferred into a sample vial with a pierceable cap and subjected to high-performance liquid chromatography using the VARIANT II β-thalassemia Short Program (Biorad). Hemoglobin composition was analyzed based on retention time and quantified relative to total hemoglobin.

### Statistical analysis

Statistics were computed using SPSS Software version 26.0.0.0 (IBM Corp, Armonk, NY). Data were described using descriptive statistics, including mean and standard deviation (SD). Mean differences were compared using Student’s t-test. Graphs were generated using Prism Software version 9.4.1 (GraphPad, Boston, MA). A *P*-value of less than 0.05 (*P* < 0.05) indicated statistical significance.

## Results

### Selinexor accelerates terminal erythroid maturation in severe β^0^-thalassemia/HbE

To confirm that the culture system reflects ineffective erythropoiesis in severe β^0^-thalassemia/HbE, CD34^+^ HSPCs from a healthy donor, a mild case, and a severe case were differentiated in the 3-phase EDM for 14 days. Maturation was assessed by CD71 and GPA expression, which showed impaired progression in β^0^-thalassemia/HbE cells, particularly reduced accumulation in R2 (CD71^Medium^/GPA^High^) and R3 (CD71^Low^/GPA^High^) at later stages (days 12–14) (S1 Fig). The defect was most pronounced in the severe case, confirming that this system faithfully recapitulates delayed terminal maturation characteristic of ineffective erythropoiesis. Therefore, this erythroid culture system can be used for downstream experiments. Moreover, the expression levels of the three key factors of interest—XPO1, HSP70, and GATA1—were assessed during in vitro erythropoiesis. The results indicated that XPO1 was highly expressed in the cytoplasm of healthy donor and β^0^-thalassemia/HbE patients (S2A,B and S3 Figs). Notably, the accumulation of cytoplasmic XPO1 protein was greater during in vitro erythropoiesis in severe β^0^-thalassemia/HbE patient. However, the expression levels of nuclear XPO1 protein were very low, as indicated by the very faint bands observed in the nuclear extracts investigated at 5 μg. We also found that cytoplasmic HSP70 was upregulated in the later stage of in vitro erythropoiesis in severe β^0^-thalassemia/HbE. This phenomenon was not observed in mild β^0^-thalassemia/HbE or healthy donor. The translocation of HSP70 led to a relative decrease in nuclear HSP70 in severe β^0^-thalassemia/HbE. In contrast, during in vitro erythropoiesis of healthy donor, the nuclear HSP70 level increased and was maintained. The observed HSP70 translocation patterns in healthy donor and severe β^0^-thalassemia/HbE patient were consistent with previous studies [12,13], confirming the comparable conditions for studying HSP70 trafficking in erythroid cells.

**Fig 1:**
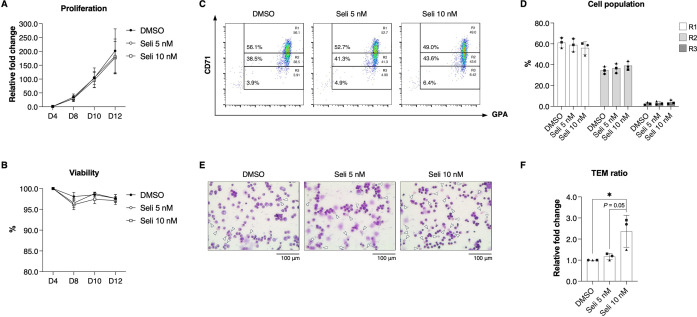
Selinexor induces terminal erythroid maturation in severe β^0^-thalassemia/HbE erythroblasts. **(A)** Cell proliferation and **(B)** cell viability after selinexor treatment during in vitro erythropoiesis. **(C)** Representative flow cytometry analysis after selinexor treatment (Seli) on culture day 14. **(D)** The percentage of cells in the gated R1 (CD71^High^/GPA^High^), R2 (CD71^Medium^/GPA^High^), and R3 (CD71^Low^/GPA^High^) populations. **(E)** Representative cell morphology after Wright-Giemsa staining on culture day 14. Arrowheads indicate enucleated erythroid cells. **(F)** Relative terminal erythroid maturation (TEM) ratio compared to the DMSO control. Data are presented as mean ± SD of three biological replicates (n = 3). *P < 0.05.

HSPCs derived from a severe β^0^-thalassemia/HbE patient were treated with each compound on day 4 and maintained throughout in vitro erythropoiesis at different concentrations. The results revealed cytotoxic effects on erythroid precursors from severe β^0^-thalassemia/HbE at higher concentrations, indicated by markedly either reduced cell proliferation or reduced cell viability of greater than 80%, including curcumin > 500 nM, oridonin > 250 nM, piperlongumine > 100 nM, plumbagin > 250 nM, KPT-185 > 5 nM, KPT-276 > 1 nM, selinexor > 10 nM, verdinexor > 1 nM, and eltanexor > 10 nM (S4 and S5 Figs). Among these nine XPO1 inhibitors, 10 nM selinexor was the most effective in improving terminal erythroid maturation, as indicated by the increased accumulation of cells in the R3 (CD71^Low^/GPA^High^) population (S6 and S7 Figs). The effect of selinexor was further examined in three independent severe β^0^-thalassemia/HbE during in vitro erythropoiesis. The results demonstrated that selinexor exhibited no cytotoxicity (Figs 1A and 1B). Additionally, treatment with 10 nM selinexor induced terminal erythroid maturation, as evidenced by an increase in late-stage erythroid cells (Figs 1C-F and S2 and S3 Table).

### Selinexor does not promote erythroid maturation via HbF production

**Fig 2:**
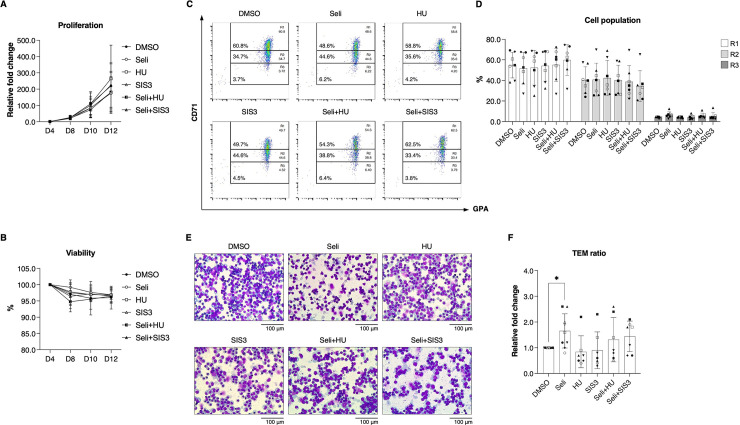
Selinexor demonstrates a dominant effect on inducing terminal erythroid maturation compared to hydroxyurea and SMAD3 inhibitor in severe β^0^-thalassemia/HbE erythroblasts. **(A)** Cell proliferation and **(B)** cell viability after single treatment with Seli (10 nM selinexor; n = 9), HU (1 μM hydroxyurea; n = 7), or SIS3 (10 nM SMAD3 inhibitor; n = 7), and combinatorial treatment (n = 7) during in vitro erythropoiesis. **(C)** Representative flow cytometry analysis on culture day 14. **(D)** The percentage of cells in the gated R1 (CD71^High^/GPA^High^), R2 (CD71^Medium^/GPA^High^), and R3 (CD71^Low^/GPA^High^) populations. **(E)** Cell morphology after Wright-Giemsa staining on culture day 14. **(F)** Relative terminal erythroid maturation (TEM) ratio compared to the DMSO control. Data are presented as mean ± SD of at least seven biological replicates. *P < 0.05.

**Fig 3:**
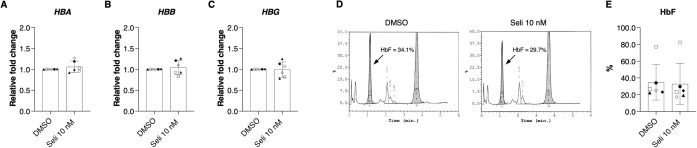
Selinexor does not alter globin mRNA and HbF expression levels in severe β^0^-thalassemia/HbE erythroblasts. Relative mRNA expression of **(A)** α-globin (*HBA*), **(B)** β-globin (*HBB*), and **(C)** γ-globin (*HBG*) normalized to the mRNA expression of *RPS18* on culture day 10 (n = 6). **(D)** Representative hemoglobin analysis on culture day 14 by HPLC. **(E)** Percentage of HbF expression levels. Data are presented as mean ± SD of six biological replicates.

Effects of selinexor, hydroxyurea (a well-known HbF-inducing compound), and SIS3 (SMAD3 inhibitor) were compared on seven independent severe β^0^-thalassemia/HbE erythroid maturation. At the concentration used, no cytotoxic effects of 1 μM hydroxyurea [20] and 10 nM SIS3 were observed (Figs 2A and 1B and S8 Fig). Our results revealed that hydroxyurea and SIS3 were unable to significantly rescue erythroid maturation in severe β^0^-thalassemia/HbE compared to selinexor (Figs 2C–F and S2 and S3 Table). Furthermore, combinatorial treatments of selinexor, hydroxyurea, and SIS3 were performed. Among these, selinexor demonstrated a dominant effect (Figs 2C–F and S2 and S3 Table), suggesting its superior efficacy in improving terminal erythroid maturation compared to hydroxyurea and SIS3 in this culture system. γ-Globin mRNA expression and HbF levels were also quantified following selinexor treatments. The results indicated that selinexor did not alter globin mRNA expression and HbF levels, suggesting an independent mechanism for improving erythroid maturation distinct from HbF induction (Figs 3A–E).

### Selinexor mediates XPO1 downregulation and cytoplasmic HSP70 accumulation

**Fig 4:**
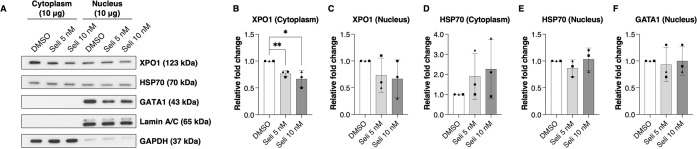
Selinexor modulates XPO1 and HSP70 expression in severe β^0^-thalassemia/HbE erythroblasts. **(A)** Representative western blot analysis on culture day 10. **(B-F)** Relative cytoplasmic and nuclear protein expression normalized to the expression of GAPDH and Lamin A/C, respectively (n = 3). Data are presented as mean ± SD of three biological replicates. *P < 0.05, **P < 0.005.

**Fig 5:**
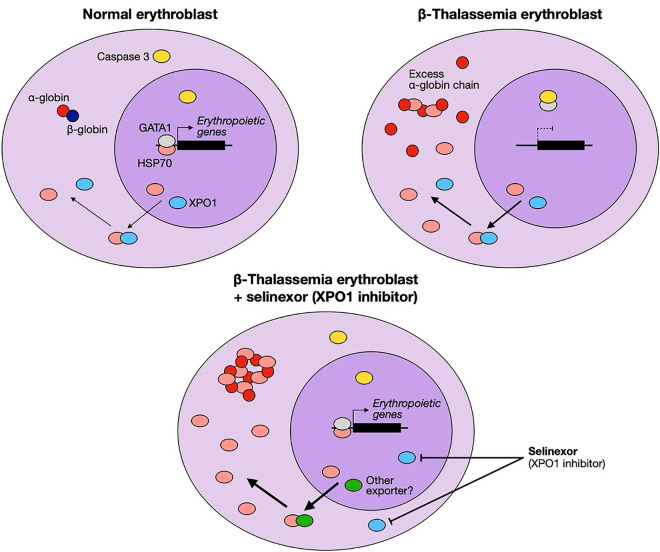
Schematic illustration of XPO1 inhibition mediated by selinexor. In normal erythroblasts, HSP70 translocation is primarily mediated by XPO1. Nuclear HSP70 protects GATA1 from caspase3-mediated degradation, thereby enabling erythropoietic gene expression and supporting normal erythroid maturation. In β-thalassemia erythroblasts, however, excess α-globin sequesters HSP70 in the cytoplasm, reducing nuclear HSP70 levels. This renders GATA1 vulnerable to caspase3-mediated degradation, leading to impaired erythropoietic gene expression and defective erythroid maturation. XPO1 inhibition by selinexor in β-thalassemia erythroblasts may promote compensatory HSP70 trafficking through alternative export pathways, resulting in increased cytoplasmic HSP70 without compromising nuclear HSP70. This dual effect may mitigate α-globin toxicity and improve erythroid maturation.

Selinexor is a well-known first-generation XPO1-selective inhibitor of nuclear export. Therefore, its effects on XPO1 downstream effectors were examined. In erythroid cells, the XPO1-HSP70-GATA1 regulatory axis has been demonstrated as an important pathway involved in the erythroid maturation process [12]. Consequently, the alteration of XPO1, HSP70, and GATA1 expression levels was investigated following selinexor treatment in erythroid precursors from severe β^0^-thalassemia/HbE patients. Here, we increased the concentration of nuclear extracts to 10 μg to accurately estimate the expression of nuclear XPO1. The results revealed that selinexor treatment inhibited XPO1 accumulation in both the cytoplasm and nucleus of severe β^0^-thalassemia/HbE erythroid precursors (Figs 4A–C and S9 Fig). This XPO1 inhibitory effect mediated by selinexor was consistent with previous studies in non-erythroid cells [22–24]. HSP70 localization was next investigated after selinexor treatment. Pharmacological inhibition of XPO1 with selinexor led to enhanced accumulation of cytoplasmic HSP70 in erythroid precursors derived from patients with severe β^0^-thalassemia/HbE (Figs 4A and 4D and S9 Fig). In contrast, nuclear HSP70 and GATA1 levels remained unchanged (Figs 4A,E–F and S9 Fig). This differs from a previous report showing that XPO1 downregulation promoted nuclear translocation of HSP70 in erythroid progenitors from healthy and β-thalassemia cells [14]. Rather than nuclear enrichment, our data demonstrate cytoplasmic accumulation of HSP70, which was associated with improved terminal erythroid maturation in severe β^0^-thalassemia/HbE. These findings suggest that erythroid maturation may be supported not only by nuclear HSP70 and GATA1 stabilization but also by increased cytoplasmic HSP70. Although XPO1 is recognized as the principal nuclear exporter of HSP70 in erythroid cells, our results indicate that HSP70 trafficking may involve additional mechanisms, as XPO1 inhibition alone did not fully redirect HSP70 to the nucleus but instead promoted cytoplasmic retention, particularly in severe β^0^-thalassemia/HbE erythroid precursors. The improvement in terminal erythroid maturation observed in severe β^0^-thalassemia/HbE may therefore be attributed, at least in part, to cytoplasmic HSP70 acting as a chaperone for excess α-globin chains. However, given the limited sample size in this study, larger cohorts will be necessary to enable more precise and in-depth analyses. A schematic summarizing the molecular mechanisms mediated by selinexor through XPO1 downregulation is presented in Fig 5.

## Discussion

XPO1-HSP70-GATA1 axis has recently been introduced as an additional regulatory pathway in erythropoiesis [12,13,25]. HSP70 is predominantly localized in the cytoplasm under basal conditions but translocates to the nucleus in response to stress stimuli such as heat shock, hypoxia, or increased reactive oxygen species [26,27]. In erythroid cells, HSP70 functions as a molecular chaperone that dynamically shuttles between the cytoplasm and nucleus through the nuclear export protein XPO1 [14,25]. Our study identifies a potentially distinct role for cytoplasmic HSP70 in erythropoiesis that extends beyond its well-established nuclear function. Traditionally, HSP70 translocates to the nucleus, where it protects the master erythroid transcription factor GATA1 from caspase3-mediated degradation, thereby safeguarding terminal erythroid maturation [12]. In β-thalassemia, however, excess unpaired α-globin chains sequester HSP70 in the cytoplasm, reducing its nuclear availability and leaving GATA1 vulnerable to degradation, a process that drives ineffective erythropoiesis [13]. Previous work demonstrated that pharmacological inhibition of XPO1 can restore nuclear retention of HSP70, stabilize GATA1, and improve erythroid differentiation [14], positioning HSP70 trafficking as a promising therapeutic axis.

In contrast, our findings suggest that the regulation of HSP70 trafficking may be more complex than previously appreciated. Selinexor, an FDA-approved XPO1 inhibitor, improved terminal erythroid maturation in severe β^0^-thalassemia/HbE progenitors; however, this effect was not associated with increased nuclear accumulation of HSP70 or GATA1. Instead, we observed a cytoplasmic accumulation of HSP70 without changes in nuclear localization. These results indicate that XPO1 inhibition does not fully redirect HSP70 to the nucleus in this disease context, and that cytoplasmic HSP70 itself may exert a beneficial function during erythropoiesis.

One possible explanation lies in the underlying pathophysiology of the disease models. β^0^-thalassemia major, most often caused by homozygous β^0^-thalassemia and examined in the previous study [14], is associated with a greater burden of cytoplasmic α-globin, leading to more pronounced depletion of nuclear HSP70 and thus a more evident nuclear rescue following XPO1 inhibition. In contrast, β^0^-thalassemia/HbE erythroid cells, used in our study, harbor a relatively lower α-globin excess, which may lessen the impact of XPO1 inhibition on nuclear HSP70. Methodological differences may also contribute. The previous study investigated CD36^+^ erythroblasts [14], representing a more immature population [28], whereas our experiments were performed on day-10 cultures enriched for basophilic and early polychromatic erythroblasts. At these more advanced stages of differentiation, nuclear activity is declining as cells prepare for enucleation, which may explain why nuclear HSP70 and GATA1 levels remained unchanged in our system.

Our results further demonstrate that selinexor did not affect HbF levels, suggesting that its effect on terminal erythroid maturation is independent of improved γ-globin expression or HbF production. Given that HbF induction has been a major therapeutic avenue in β-thalassemia, we hypothesized that combining selinexor with other compounds might yield additive or synergistic benefits. However, single-compound treatments with hydroxyurea or SIS3 , as well as co-treatments with selinexor, did not further enhance terminal erythroid maturation. These findings underscore the strong standalone efficacy of selinexor in ameliorating ineffective erythropoiesis and support its potential use as a modifying agent in β-thalassemia.

Together, our data raise the possibility that cytoplasmic HSP70 may act as a functional chaperone for excess α-globin, reducing proteotoxic stress in a manner analogous to α-hemoglobin stabilizing protein (AHSP). This protective role could explain the improvement in terminal erythroid maturation observed following selinexor treatment, despite unchanged nuclear HSP70 and GATA1 levels. Moreover, our observations suggest that XPO1 inhibition alone does not fully block HSP70 trafficking, as HSP70 translocation persisted even with reduced XPO1 levels. This points to the involvement of additional transport pathways. It is also important to recognize that XPO1 exports a wide range of proteins and oligonucleotides [29], raising the possibility that the improvement in terminal erythroid differentiation observed with selinexor may, at least in part, reflect off-target effects. This warrants further investigation to delineate the specific contribution of XPO1-HSP70 modulation versus potential off-target mechanisms. Indeed, the previous study has shown that phosphorylation of HSP27 can drive its nuclear entry in late erythroblasts, where it competes with HSP70 and promotes GATA1 degradation, thereby facilitating terminal differentiation [30]. Such findings highlight the complexity of heat-shock protein dynamics and suggest that multiple regulatory mechanisms converge to shape erythroid maturation.

In conclusion, our results broaden the understanding of HSP70 biology in β-thalassemia by revealing a potential cytoplasmic role in mitigating α-globin toxicity, independent of HbF induction. These findings suggest that therapeutic strategies targeting HSP70 trafficking may need to consider both nuclear and cytoplasmic functions. Further studies dissecting the precise molecular partners and transport mechanisms of HSP70, as well as in vivo validation of selinexor, will be essential to fully establish its therapeutic potential in β-thalassemia.

## Supporting information

S1 Fig Flow cytometry analysis of CD71 and GPA expression during in vitro erythropoiesis using the 3-phase erythroid differentiation medium. Healthy donor (Normal; n = 1); mild β^0^-thalassemia/HbE (β^0^/E-Mild; n = 1); severe β^0^-thalassemia/HbE (β^0^/E-Severe; n = 1).
(PDF)

S2 Fig Expression levels of XPO1, HSP70, and GATA1 during in vitro erythropoiesis using the 3-phase erythroid differentiation medium. (A) Western blot analysis of cytoplasmic and nuclear extracts. (B) Plots generated by ImageJ showing the fold change in expression levels. Data were normalized to the loading control bands and expressed relative to day 8. Healthy donor (Normal; n = 1); mild β^0^-thalassemia/HbE (β^0^/E-Mild; n = 1); severe β^0^-thalassemia/HbE (β^0^/E-Severe; n = 1).
(PDF)

S3 Fig Expression of XPO1, HSP70, GATA1, Lamin A/C, and GAPDH during in vitro erythropoiesis. Uncropped X-ray films show the expression of XPO1, HSP70, GATA1, Lamin A/C, and GAPDH in erythroid progenitors from a healthy donor (Normal; n = 1), mild β^0^-thalassemia/HbE (β^0^/E-Mild; n = 1), and severe β^0^-thalassemia/HbE (β^0^/E-Severe; n = 1). Cropping areas are indicated by black rectangles.
(PDF)

S4 Fig Screening of nine XPO1 inhibitors on cell proliferation. The effects of nine XPO1 inhibitors on proliferation of erythroid progenitors from a severe β^0^-thalassemia/HbE patient (n = 1) during in vitro erythropoiesis. All data were obtained from one patient, who yielded the highest number of extracted CD34 ⁺ HSPCs.
(PDF)

S5 Fig Screening of nine XPO1 inhibitors on cell viability. The effects of nine XPO1 inhibitors on viability of erythroid progenitors from a severe β^0^-thalassemia/HbE patient (n = 1) during in vitro erythropoiesis. All data were obtained from one patient, who yielded the highest number of extracted CD34 ⁺ HSPCs.
(PDF)

S6 Fig Screening of five synthetic XPO1 inhibitors on erythroid cell differentiation. Flow cytometry analysis of erythroid progenitors from a severe β^0^-thalassemia/HbE patient (n = 1) treated with five synthetic XPO1 inhibitors on culture day 14. All data were obtained from one patient, who yielded the highest number of extracted CD34 ⁺ HSPCs.
(PDF)

S7 Fig Screening of four natural XPO1 inhibitors on erythroid cell differentiation. Flow cytometry analysis of erythroid progenitors from a severe β^0^-thalassemia/HbE patient (n = 1) treated with four natural XPO1 inhibitors on culture day 14. All data were obtained from one patient, who yielded the highest number of extracted CD34 ⁺ HSPCs.
(PDF)

S8 Fig Screening of the SMAD3 inhibitor (SIS3) on cell proliferation and viability. The effects of SIS3 on proliferation and viability of erythroid progenitors from a severe β^0^-thalassemia/HbE patient (n = 1) during in vitro erythropoiesis. All data were obtained from one patient, who yielded the highest number of extracted CD34^+^ HSPCs.
(PDF)

S9 Fig Expression of XPO1, HSP70, GATA1, Lamin A/C, and GAPDH after selinexor treatment. Uncropped X-ray films show the expression of XPO1, HSP70, GATA1, Lamin A/C, and GAPDH in erythroid progenitors from severe β^0^-thalassemia/HbE patients (n = 3) following selinexor treatment. Cropping areas are indicated by black rectangles.
(PDF)

S1 Table Baseline demographic and clinical characteristics of severe β^0^-thalassemia/HbE patients participating in this study. (PDF)

S2 Table Erythroid cell counts assessed in triplicate. (PDF)

S3 Table Differential counts of erythroid cells. (PDF)

S4 Table Primers used in this study. (PDF)
