# Supplementary material for: Inhibition of XPO1 by selinexor enhances terminal erythroid maturation through modulation of HSP70 trafficking in severe β0-thalassemia/HbE
Source: PLoS One. 2025 Sep 25;20(9):e0333127. doi: 10.1371/journal.pone.0333127 (PMC12463213; doi:10.1371/journal.pone.0333127)
Supplement: S1 Fig — Healthy donor (Normal; n = 1); mild β0-thalassemia/HbE (β0/E-Mild; n = 1); severe β0-thalassemia/HbE (β0/E-Severe; n = 1). (PDF) [file pone.0333127.s001.pdf]

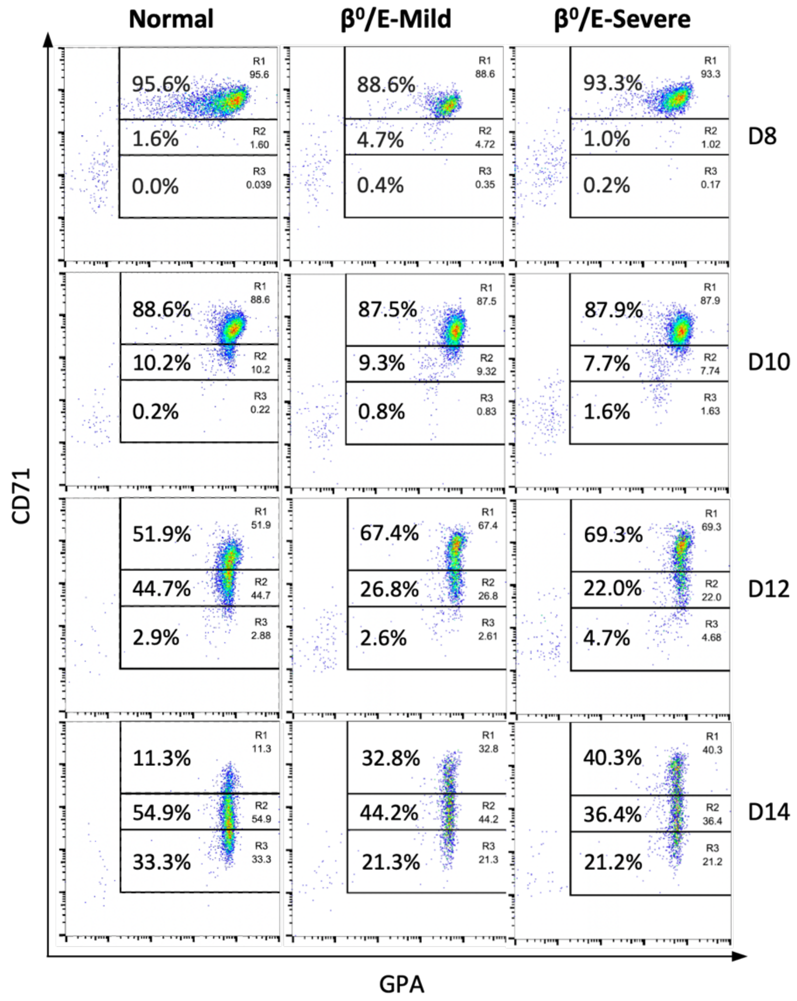

S1 Fig. Flow cytometry analysis of CD71 and GPA expression during in vitro erythropoiesis using the 3-phase erythroid differentiation medium. Healthy donor (Normal; n=1); mild β^0-thalassemia/HbE (β^0/E-Mild; n=1); severe β^0-thalassemia/HbE (β^0/E-Severe; n=1).
