# Supplementary material for: Inhibition of XPO1 by selinexor enhances terminal erythroid maturation through modulation of HSP70 trafficking in severe β0-thalassemia/HbE
Source: PLoS One. 2025 Sep 25;20(9):e0333127. doi: 10.1371/journal.pone.0333127 (PMC12463213; doi:10.1371/journal.pone.0333127)
Supplement: S2 Fig — (A) Western blot analysis of cytoplasmic and nuclear extracts. (B) Plots generated by ImageJ showing the fold change in expression levels. Data were normalized to the loading control bands and expressed relative to day 8. Healthy donor (Normal; n = 1); mild β0-thalassemia/HbE (β0/E-Mild; n = 1); severe β0-thalassemia/HbE (β0/E-Severe; n = 1). (PDF) [file pone.0333127.s002.pdf]

A

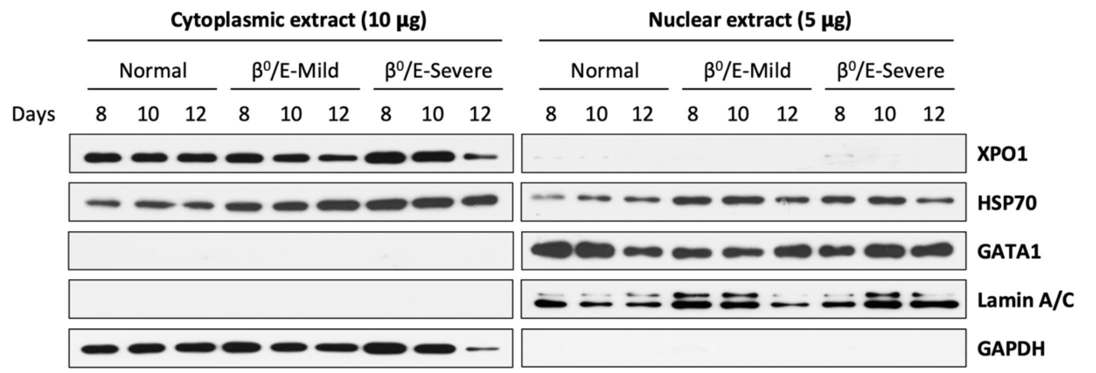

B

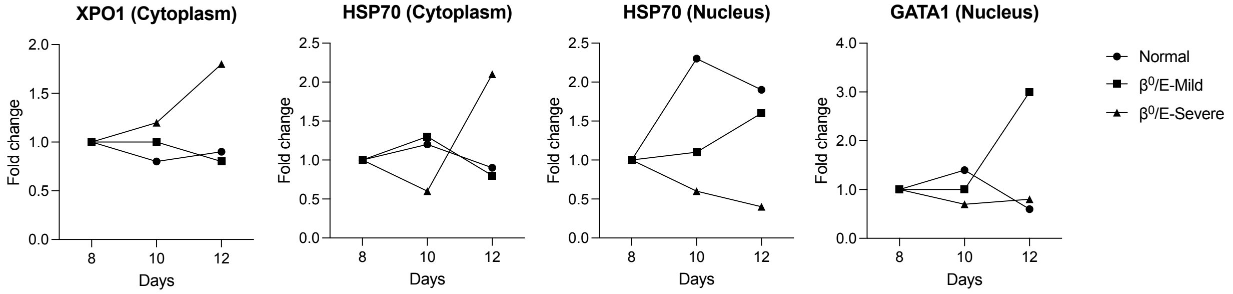

S2 Fig. Expression levels of XPO1, HSP70, and GATA1 during in vitro erythropoiesis using the 3-phase erythroid differentiation medium. (A) Western blot analysis of cytoplasmic and nuclear extracts. (B) Plots generated by ImageJ showing the fold change in expression levels. Data were normalized to the loading control bands and expressed relative to day 8. Healthy donor (Normal; n=1); mild β^0-thalassemia/HbE (β^0/E-Mild; n=1); severe β^0-thalassemia/HbE (β^0/E-Severe; n=1).
