# Supplementary material for: Inhibition of XPO1 by selinexor enhances terminal erythroid maturation through modulation of HSP70 trafficking in severe β0-thalassemia/HbE
Source: PLoS One. 2025 Sep 25;20(9):e0333127. doi: 10.1371/journal.pone.0333127 (PMC12463213; doi:10.1371/journal.pone.0333127)
Supplement: S3 Fig — Uncropped X-ray films show the expression of XPO1, HSP70, GATA1, Lamin A/C, and GAPDH in erythroid progenitors from a healthy donor (Normal; n = 1), mild β0-thalassemia/HbE (β0/E-Mild; n = 1), and severe β0-thalassemia/HbE (β0/E-Severe; n = 1). Cropping areas are indicated by black rectangles. (PDF) [file pone.0333127.s003.pdf]

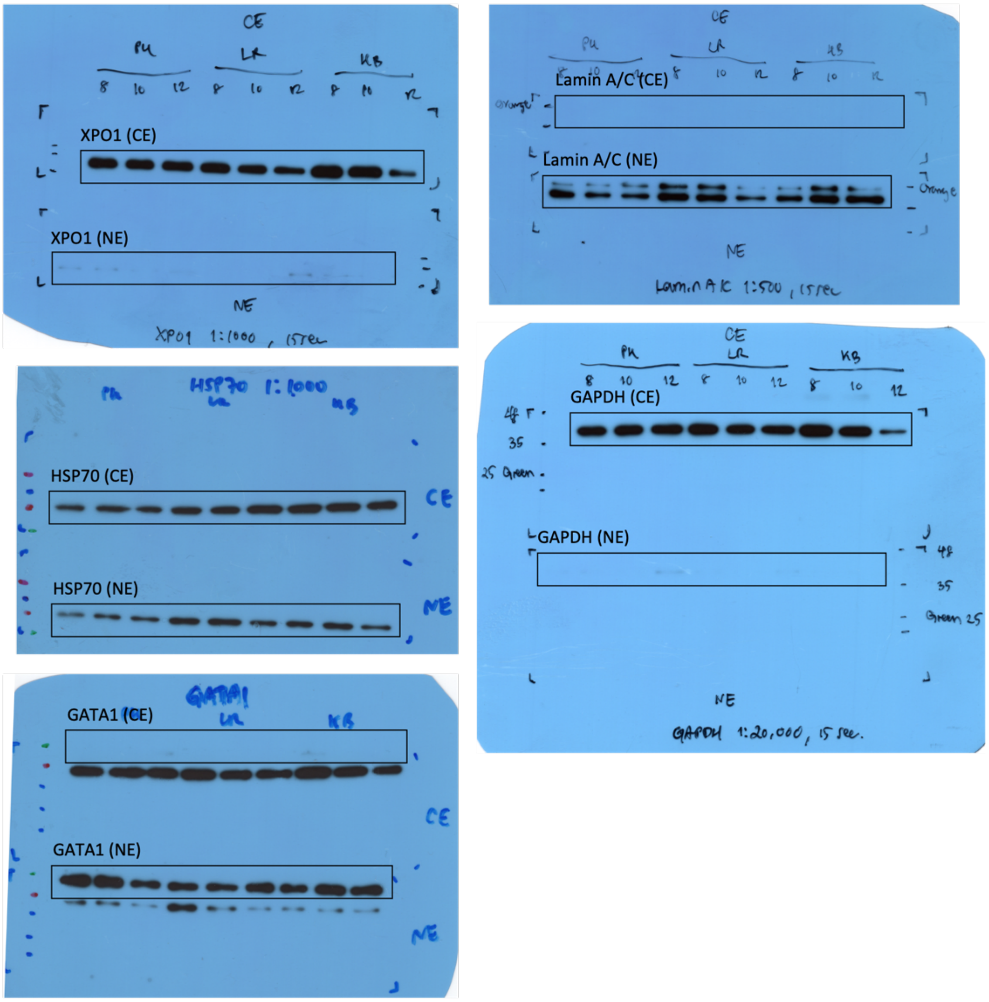

S3 Fig. Expression of XPO1, HSP70, GATA1, Lamin A/C, and GAPDH during in vitro erythropoiesis. Uncropped X-ray films show the expression of XPO1, HSP70, GATA1, Lamin A/C, and GAPDH in erythroid progenitors from a healthy donor (Normal; n=1), mild β^0-thalassemia/HbE (β^0/E-Mild; n=1), and severe β^0-thalassemia/HbE (β^0/E-Severe; n=1). Cropping areas are indicated by black rectangles.
