# Supplementary material for: Inhibition of XPO1 by selinexor enhances terminal erythroid maturation through modulation of HSP70 trafficking in severe β0-thalassemia/HbE
Source: PLoS One. 2025 Sep 25;20(9):e0333127. doi: 10.1371/journal.pone.0333127 (PMC12463213; doi:10.1371/journal.pone.0333127)
Supplement: S7 Fig — Flow cytometry analysis of erythroid progenitors from a severe β0-thalassemia/HbE patient (n = 1) treated with four natural XPO1 inhibitors on culture day 14. All data were obtained from one patient, who yielded the highest number of extracted CD34 ⁺ HSPCs. (PDF) [file pone.0333127.s007.pdf]

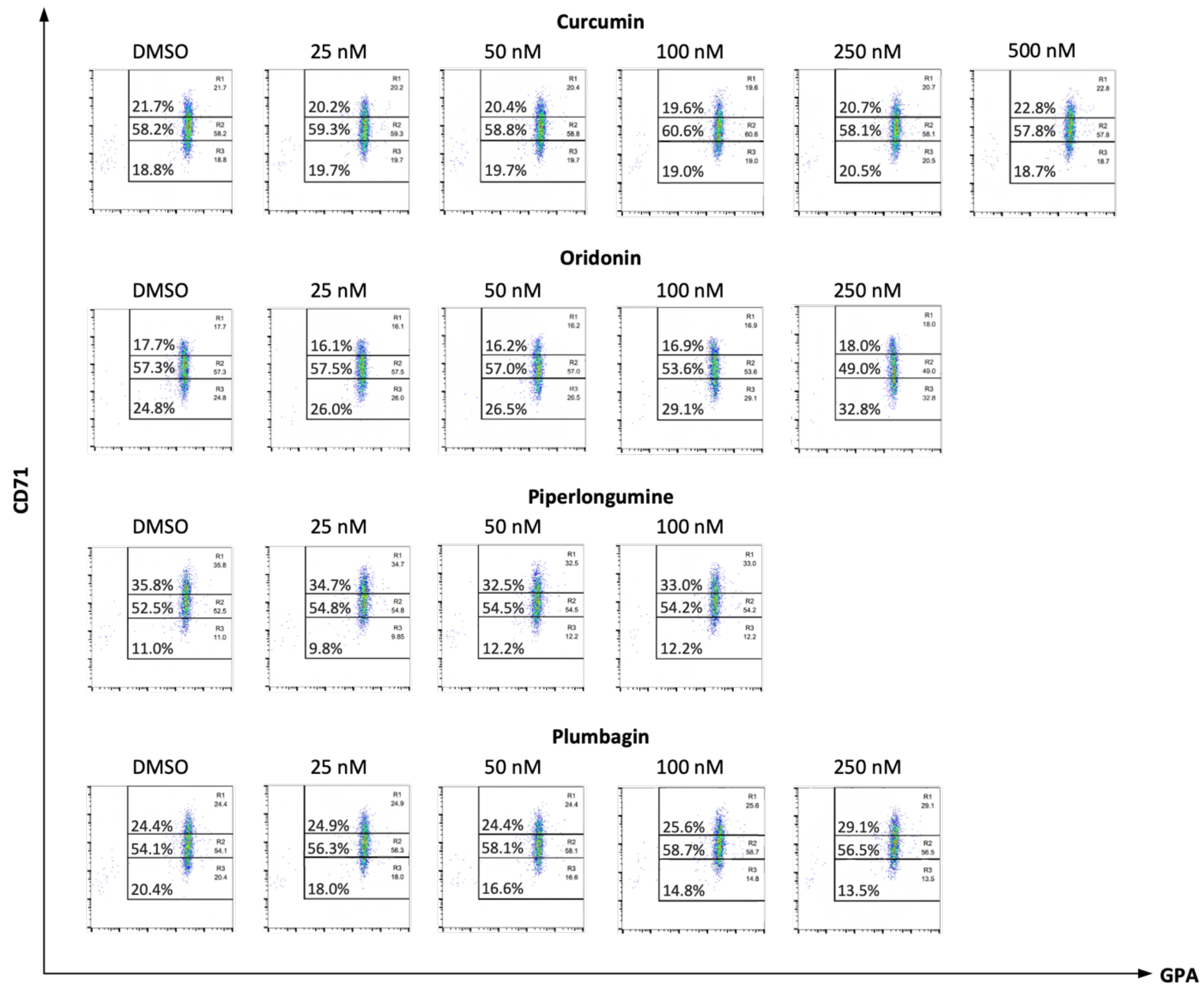

S7 Fig. Screening of four natural XPO1 inhibitors on erythroid cell differentiation. Flow cytometry analysis of erythroid progenitors from a severe β^0-thalassemia/HbE patient (n=1) treated with four natural XPO1 inhibitors on culture day 14. All data were obtained from one patient, who yielded the highest number of extracted $CD34^+$ HSPCs.
