# Supplementary material for: Inhibition of XPO1 by selinexor enhances terminal erythroid maturation through modulation of HSP70 trafficking in severe β0-thalassemia/HbE
Source: PLoS One. 2025 Sep 25;20(9):e0333127. doi: 10.1371/journal.pone.0333127 (PMC12463213; doi:10.1371/journal.pone.0333127)
Supplement: S9 Fig — Uncropped X-ray films show the expression of XPO1, HSP70, GATA1, Lamin A/C, and GAPDH in erythroid progenitors from severe β0-thalassemia/HbE patients (n = 3) following selinexor treatment. Cropping areas are indicated by black rectangles. (PDF) [file pone.0333127.s009.pdf]

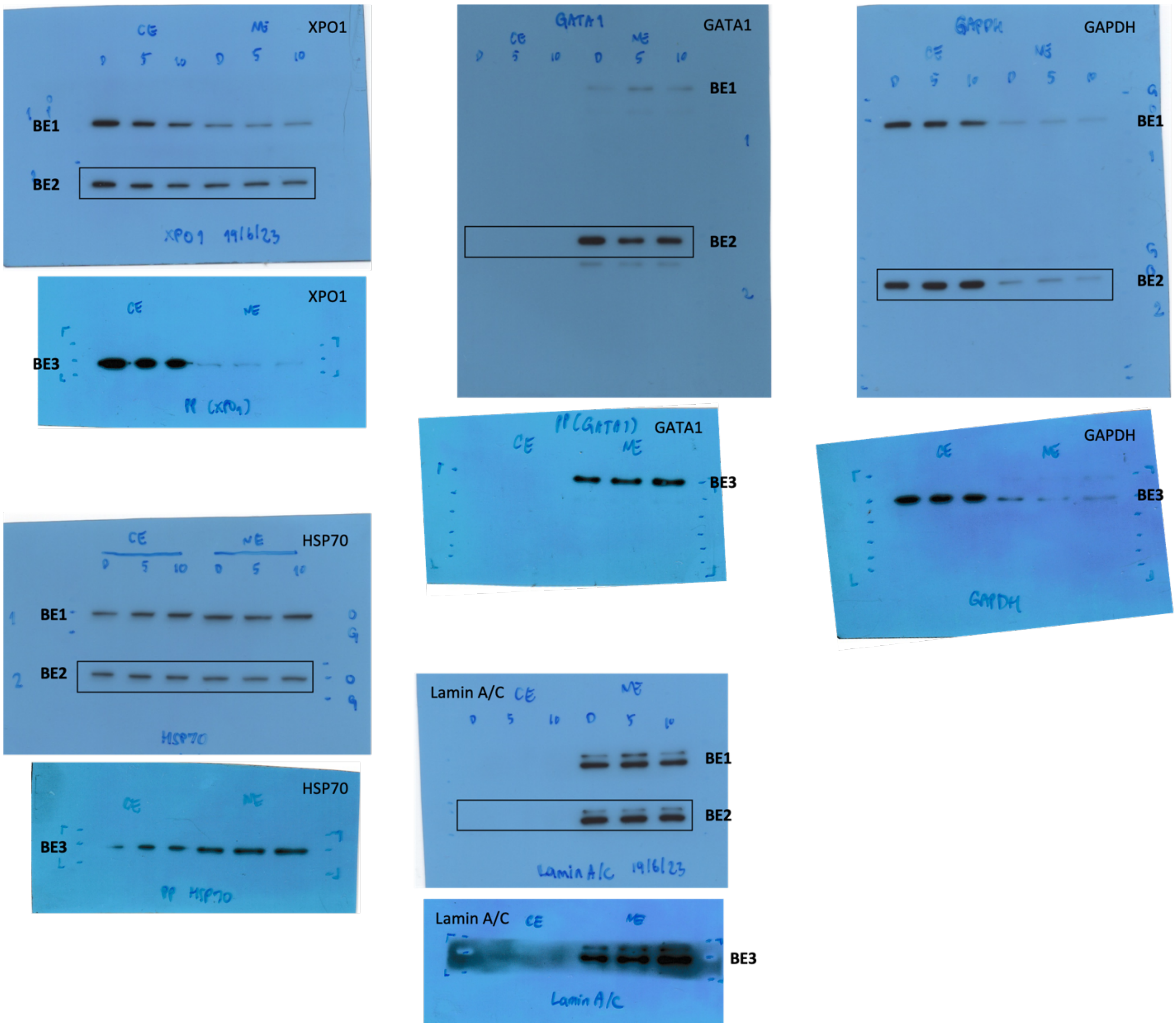

S9 Fig. Expression of XPO1, HSP70, GATA1, Lamin A/C, and GAPDH after selinexor treatment. Uncropped X-ray films show the expression of XPO1, HSP70, GATA1, Lamin A/C, and GAPDH in erythroid progenitors from severe β^0-thalassemia/HbE patients (n=3) following selinexor treatment. Cropping areas are indicated by black rectangles.
