# Supplementary material for: Inhibition of XPO1 by selinexor enhances terminal erythroid maturation through modulation of HSP70 trafficking in severe β0-thalassemia/HbE
Source: PLoS One. 2025 Sep 25;20(9):e0333127. doi: 10.1371/journal.pone.0333127 (PMC12463213; doi:10.1371/journal.pone.0333127)
Supplement: S1 Table — (PDF) [file pone.0333127.s010.pdf]

S1 Table. Baseline demographic and clinical characteristics of severe β^0-thalassemia/HbE patients participating in this study.

Characteristic	Total (n=13)
Age – year	
Mean ± SD	28.0 ± 7.4
Female – no. (%)	7 (53.8)
Genotype – no. (%)	
CD41/42 (-TCTT) and CD26 (G>A)	6 (46.1)
CD17 (A>T) and CD26 (G>A)	4 (30.8)
CD71/72 (+A) and CD26 (G>A)	2 (15.4)
IVSI-1 (G>T) and CD26 (G>A)	1 (7.7)
RBC – 10^6/µL	
Mean ± SD	3.3 ± 0.4
Hb – g/dL*	
Mean ± SD	6.6 ± 0.7
Hct – %	
Mean ± SD	21.2 ± 2.5
MCV – fL	
Mean ± SD	65.6 ± 6.7
MCH – pg	
Mean ± SD	20.5 ± 1.6
HbA_2+HbE – %†	
Mean ± SD	68.5 ± 12.2
HbF – %	
Mean ± SD	31.5 ± 12.2

*, Hb was determined at steady state or before receiving a blood transfusion.

†, HbA_2 and HbE were coeluted following HPLC.
