# Supplementary material for: Inhibition of XPO1 by selinexor enhances terminal erythroid maturation through modulation of HSP70 trafficking in severe β0-thalassemia/HbE
Source: PLoS One. 2025 Sep 25;20(9):e0333127. doi: 10.1371/journal.pone.0333127 (PMC12463213; doi:10.1371/journal.pone.0333127)
Supplement: S2 Table — (PDF) [file pone.0333127.s011.pdf]

S2 Table. Erythroid cell counts assessed in triplicate.

Patient	Basophilic erythroblast	Polychromatic erythroblast	Orthochromatic erythroblast	Enucleated erythroid cell
1				
DMSO	3,2,4	8,7,6	78,78,77	11,13,13
Seli 5 nM	2,2,3	5,6,7	78,76,78	15,16,12
Seli 10 nM	1,1,0	4,3,3	81,83,85	14,13,12
2				
DMSO	0,0,0	5,5,4	86,85,87	9,10,9
Seli 5 nM	0,0,0	3,4,4	94,93,93	3,3,3
Seli 10 nM	0,0,0	2,1,2	94,94,92	4,5,6
3				
DMSO	0,0,0	6,5,4	69,70,68	25,25,28
Seli 5 nM	0,0,0	5,5,5	71,69,67	24,26,28
Seli 10 nM	0,0,0	3,3,4	59,60,61	38,37,35
4				
DMSO	11,11,11	32,34,32	54,52,55	3,3,2
Seli 10 nM	6,8,9	21,21,20	66,65,64	7,6,7
5				
DMSO	6,5,7	53,52,51	39,40,39	2,3,3
Seli 10 nM	4,4,3	39,39,38	51,50,50	6,7,9
6				
DMSO	10,12,11	36,36,33	52,50,54	2,2,2
Seli 10 nM	7,7,8	34,33,34	46,47,46	13,13,12
HU 1 µM	19,19,18	44,44,47	28,29,29	9,8,6

Patient	Basophilic erythroblast	Polychromatic erythroblast	Orthochromatic erythroblast	Enucleated erythroid cell
SIS3 10 nM	25,24,24	31,35,32	36,34,36	8,7,8
Seli+HU	8,9,8	51,52,52	29,28,28	12,11,12
Seli+SIS3	7,9,9	48,48,47	38,36,37	7,7,7
7				
DMSO	6,4,4	50,53,49	36,34,36	8,9,11
Seli 10 nM	3,2,3	30,28,31	44,43,43	23,27,23
HU 1 µM	4,4,5	32,34,30	49,49,50	15,13,15
SIS3 10 nM	4,5,4	31,30,30	57,60,58	8,5,8
Seli+HU	4,3,2	31,29,32	51,54,53	14,14,13
Seli+SIS3	1,0,0	38,36,36	42,42,42	19,22,22
8				
DMSO	5,5,4	43,42,44	45,44,45	7,9,7
Seli 10 nM	6,5,6	21,21,19	46,46,48	27,28,27
HU 1 µM	3,3,4	55,54,54	39,39,41	3,4,1
SIS3 10 nM	5,5,6	58,59,57	34,34,35	3,2,2
Seli+HU	2,2,1	25,23,25	50,50,50	23,25,24
Seli+SIS3	5,5,4	29,28,30	50,49,51	16,18,15
9				
DMSO	5,6,4	60,62,62	32,32,34	3,0,0
Seli 10 nM	6,4,6	57,57,56	33,35,35	4,4,3
HU 1 µM	7,7,4	66,68,68	25,24,24	2,1,4
SIS3 10 nM	1,2,1	71,69,69	27,28,26	1,1,4
Seli+HU	2,2,3	65,67,68	30,27,27	3,4,2
Seli+SIS3	5,5,2	61,58,61	33,36,36	1,1,1

Patient	Basophilic erythroblast	Polychromatic erythroblast	Orthochromatic erythroblast	Enucleated erythroid cell
10				
DMSO	0,0,0	20,24,23	68,66,66	12,10,11
Seli 10 nM	1,0,0	23,22,20	67,69,70	9,9,10
HU 1 µM	7,6,9	26,28,30	62,60,59	5,6,2
SIS3 10 nM	18,16,16	34,32,34	41,44,45	7,8,5
Seli+HU	8,8,6	17,19,21	67,63,67	8,10,6
Seli+SIS3	7,6,9	21,24,19	58,60,53	14,10,19
11				
DMSO	4,4,2	8,11,10	69,73,71	19,12,17
Seli 10 nM	0,0,0	18,17,13	68,72,73	14,11,14
HU 1 µM	9,5,11	17,18,18	56,59,55	18,18,16
SIS3 10 nM	6,3,2	24,24,26	54,53,56	16,20,16
Seli+HU	0,3,3	22,21,23	58,58,52	20,18,22
Seli+SIS3	0,0,0	5,9,8	64,65,60	31,26,32
12				
DMSO	2,1,4	19,22,21	62,63,59	17,14,16
Seli 10 nM	1,2,0	15,13,15	44,41,40	40,44,45
HU 1 µM	1,1,1	28,24,22	41,41,44	30,34,33
SIS3 10 nM	4,6,1	14,11,18	54,52,50	28,31,31
Seli+HU	2,2,1	17,15,15	46,43,46	35,40,38
Seli+SIS3	1,0,1	14,12,15	52,52,50	33,36,34
