# Supplementary material for: Inhibition of XPO1 by selinexor enhances terminal erythroid maturation through modulation of HSP70 trafficking in severe β0-thalassemia/HbE
Source: PLoS One. 2025 Sep 25;20(9):e0333127. doi: 10.1371/journal.pone.0333127 (PMC12463213; doi:10.1371/journal.pone.0333127)
Supplement: S3 Table — (PDF) [file pone.0333127.s012.pdf]

S3 Table. Differential counts of erythroid cells.

Patient	Basophilic erythroblast (%)	Polychromatic erythroblast (%)	Orthochromatic erythroblast (%)	Enucleated erythroid cell (%)	TEM ratio*
1					
DMSO	3.0	7.0	77.7	12.3	9.0
Seli 5 nM	2.3	6.0	77.3	14.3	11.0
Seli 10 nM	0.7	3.3	83.0	13.0	24.0
2					
DMSO	0.0	4.7	86.0	9.3	20.4
Seli 5 nM	0.0	3.7	93.3	3.0	26.3
Seli 10 nM	0.0	1.7	93.3	5.0	59.0
3					
DMSO	0.0	5.0	69.0	26.0	19.0
Seli 5 nM	0.0	5.0	69.0	26.0	19.0
Seli 10 nM	0.0	3.3	60.0	36.7	29.0
4					
DMSO	11.0	32.7	53.7	2.7	1.3
Seli 10 nM	7.7	20.7	65.0	6.7	2.5
5					
DMSO	6.0	52.0	39.3	2.7	0.7
Seli 10 nM	3.7	38.7	50.3	7.3	1.4
6					
DMSO	11.0	35.0	52.0	2.0	1.2
Seli 10 nM	7.3	33.7	46.3	12.7	1.4
HU 1 μM	18.7	45.0	28.7	7.7	0.6

Patient	Basophilic erythroblast (%)	Polychromatic erythroblast (%)	Orthochromatic erythroblast (%)	Enucleated erythroid cell (%)	TEM ratio*
SIS3 10 nM	24.3	32.7	35.3	7.7	0.8
Seli+HU	8.3	51.7	28.3	11.7	0.7
Seli+SIS3	8.3	47.7	37.0	7.0	0.8
7					
DMSO	4.7	50.7	35.3	9.3	0.8
Seli 10 nM	2.7	29.7	43.3	24.3	2.1
HU 1 μM	4.3	32.0	49.3	14.3	1.8
SIS3 10 nM	4.3	30.3	58.3	7.0	1.9
Seli+HU	3.0	30.7	52.7	13.7	2.0
Seli+SIS3	0.3	36.7	42.0	21.0	1.7
8					
DMSO	4.7	43.0	44.7	7.7	1.1
Seli 10 nM	5.7	20.3	46.7	27.3	2.8
HU 1 μM	3.3	54.3	39.7	2.7	0.7
SIS3 10 nM	5.3	58.0	34.3	2.3	0.6
Seli+HU	1.7	24.3	50.0	24.0	2.8
Seli+SIS3	4.7	29.0	50.0	16.3	2.0
9					
DMSO	5.0	61.3	32.7	1.0	0.5
Seli 10 nM	5.3	56.7	34.3	3.7	0.6
HU 1 μM	6.0	67.3	24.3	2.3	0.4
SIS3 10 nM	1.3	69.7	27.0	2.0	0.4
Seli+HU	2.3	66.7	28.0	3.0	0.4
Seli+SIS3	4.0	60.0	35.0	1.0	0.6

Patient	Basophilic erythroblast (%)	Polychromatic erythroblast (%)	Orthochromatic erythroblast (%)	Enucleated erythroid cell (%)	TEM ratio*
10					
DMSO	0.0	22.3	66.7	11.0	3.5
Seli 10 nM	0.3	21.7	68.7	9.3	3.5
HU 1 µM	7.3	28.0	60.3	4.3	1.8
SIS3 10 nM	16.7	33.3	43.3	6.7	1.0
Seli+HU	7.3	19.0	65.7	8.0	2.8
Seli+SIS3	7.3	21.3	57.0	14.3	2.5
11					
DMSO	3.3	9.7	71.0	16.0	6.7
Seli 10 nM	0.0	16.0	71.0	13.0	5.3
HU 1 µM	8.3	17.7	56.7	17.3	2.8
SIS3 10 nM	3.7	24.7	54.3	17.3	2.5
Seli+HU	2.0	22.0	56.0	20.0	3.2
Seli+SIS3	0.0	7.3	63.0	29.7	12.6
12					
DMSO	2.3	20.7	61.3	15.7	3.3
Seli 10 nM	1.0	14.3	41.7	43.0	5.5
HU 1 µM	1.0	24.7	42.0	32.3	2.9
SIS3 10 nM	3.7	14.3	52.0	30.0	4.6
Seli+HU	1.7	15.7	45.0	37.7	4.8
Seli+SIS3	0.7	13.7	51.3	34.3	6.0

*TEM ratio, terminal erythroid maturation ratio
