# Supplementary material for: Inhibition of XPO1 by selinexor enhances terminal erythroid maturation through modulation of HSP70 trafficking in severe β0-thalassemia/HbE
Source: PLoS One. 2025 Sep 25;20(9):e0333127. doi: 10.1371/journal.pone.0333127 (PMC12463213; doi:10.1371/journal.pone.0333127)
Supplement: S4 Table — (PDF) [file pone.0333127.s013.pdf]

S4 Table. Primers used in this study.

Target mRNA	Primer name	Sequence (5'-3')
HBA	HBA-F	TGG ACC CGG TCA ACT TCA AG
	HBA-R	TCA CAG AAG CCA GGA ACT TGT
HBB	HBB-F	GAA GGC TCA TGG CAA GAA AG
	HBB-R	CAC TGG TGG GGT GAA TTC TT
HBG	HBG-F	TCA CAG AGG AGG ACA AGG CTA
	HBG-R	GCT TTA TGG CAT CTC CCA AG
RPS18	RPS18-F	GGA TGA GGT GGA ACG TGT
	RPS18-R	CTA GGA CCT GGC TGT ATT TTC
